# The role of angiogenic factors in predicting clinical outcome in severe bacterial infection in Malawian children

**DOI:** 10.1186/cc9025

**Published:** 2010-05-21

**Authors:** Limangeni A Mankhambo, Daniel L Banda, The IPD Study Group, Graham Jeffers, Sarah A White, Paul Balmer, Standwell Nkhoma, Happy Phiri, Elizabeth M Molyneux, C Anthony Hart, Malcolm E Molyneux, Robert S Heyderman, Enitan D Carrol

**Affiliations:** 1 Malawi-Liverpool-Wellcome Trust Clinical Research Programme, College of Medicine, University of Malawi, Blantyre, Malawi; 2 Department of Paediatrics, College of Medicine, University of Malawi, Blantyre, Malawi; 3 Health Protection Agency, Manchester Medical Microbiology Partnership, Oxford Road, Manchester, M13 9WZ, UK; 4 Division of Child Health, The University of Liverpool, Institute of Child Health, Alder Hey Children's NHS Foundation Trust, Eaton Road, Liverpool, L12 2AP, UK; 5 Division of Medical Microbiology, The University of Liverpool, Duncan Building, Daulby Street, Liverpool, L69 3GA, UK

## Abstract

**Introduction:**

Severe sepsis is a disease of the microcirculation, with endothelial dysfunction playing a key role in its pathogenesis and subsequent associated mortality. Angiogenesis in damaged small vessels may ameliorate this dysfunction. The aim of the study was to determine whether the angiogenic factors (vascular endothelial growth factor (VEGF), platelet-derived growth factor (PDGF), fibroblast growth factor (FGF), and angiopoietin-1 (Ang-1) and -2 (Ang-2)) are mortality indicators in Malawian children with severe bacterial infection.

**Methods:**

In 293 children with severe bacterial infection, plasma VEGF, PDGF, FGF, and Ang-1 and Ang-2 were measured on admission; in 50 of the children with meningitis, VEGF, PDGF, and FGF were also measured in the CSF. Healthy controls comprised children from some of the villages of the index cases. Univariable and multivariable logistic regression analyses were performed to develop a prognostic model.

**Results:**

The median age was 2.4 years, and the IQR, 0.7 to 6.0 years. There were 211 children with bacterial meningitis (72%) and 82 (28%) with pneumonia, and 154 (53%) children were HIV infected. Mean VEGF, PDGF, and FGF concentrations were higher in survivors than in nonsurvivors, but only PDGF remained significantly increased in multivariate analysis (*P *= 0.007). Mean Ang-1 was significantly increased, and Ang-2 was significantly decreased in survivors compared with nonsurvivors (6,000 versus 3,900 pg/ml, *P *= 0.03; and 7,700 versus 11,900 pg/ml, *P *= 0.02, respectively). With a logistic regression model and controlling for confounding factors, only female sex (OR, 3.95; 95% CI, 1.33 to 11.76) and low Ang-1 (OR, 0.23; 95% CI, 0.08 to 0.69) were significantly associated with mortality. In children with bacterial meningitis, mean CSF VEGF, PDGF, and FGF concentrations were higher than paired plasma concentrations, and mean CSF, VEGF, and FGF concentrations were higher in nonsurvivors than in survivors (*P *= 0.02 and 0.001, respectively).

**Conclusions:**

Lower plasma VEGF, PDGF, FGF, and Ang-1 concentrations and higher Ang-2 concentrations are associated with an unfavorable outcome in children with severe bacterial infection. These angiogenic factors may be important in the endothelial dysregulation seen in severe bacterial infection, and they could be used as biomarkers for the early identification of patients at risk of a poor outcome.

## Introduction

Sepsis remains a leading cause of death in children in the developing world, accounting for some 60% of childhood mortality. *Streptococcus pneumoniae *and *Haemophilus influenzae *type b, two pathogens responsible for most childhood deaths of pneumonia and bacterial meningitis, caused more than a million deaths globally in children younger than 5 years in 2000 [1,2]. Severe sepsis is a disease of the microcirculation, with endothelial dysfunction playing a key role in its pathogenesis and subsequent associated mortality [3]. Endothelial progenitor cells from the bone marrow ameliorate the dysfunction caused by severe sepsis, and this process is thought to be mediated by angiogenesis in ischemic areas and in damaged small vessels [4,5].

Growth factors are recognized for their ability to induce cellular proliferation and differentiation. Vascular endothelial growth factor (VEGF), a dimeric 46-kDa glycoprotein, is an endothelial cell-specific, multifunctional cytokine. VEGF is a potent regulator of vascular permeability and angiogenesis, and in endothelial cells, induces the expression of cell-adhesion molecules and the release of cytokines and chemokines [6,7]. Platelet-derived growth factor (PDGF) has angiogenic effects and stimulates endothelial cell migration [8,9]. Despite the name "platelet derived," studies suggest that the endothelium rather than platelets might be a major source of PDGF in sepsis [10]. Fibroblast growth factor (FGF) promotes angiogenesis and also has antiapoptotic effects [11,12]. Elevated CSF levels of FGF have been observed in children with bacterial meningitis and are associated with poor outcome, suggesting neurotropic effects [13].

The angiopoietins, angiopoietin-1 (Ang-1) and angiopoietin-2 (Ang-2), play a fundamental role in the maintenance of vessel integrity. Angiopoietin-1 (Ang-1) and Ang-2 are ligands of the endothelial receptor tyrosine kinase Tie-2, which is a key regulator of endothelial function [14]. Binding of circulating Ang-1 to the Tie-2 receptor protects the vasculature from inflammation and leakage, whereas binding of Ang-2 antagonizes Tie-2 signaling and disrupts endothelial barrier function. Ang-1 is important for blood vessel stability, inhibiting vascular leakage, and suppressing inflammatory gene expression [15,16]. Ang-2 is generally an antagonist of Ang-1, but in the presence of VEGF, promotes cell survival [17]. Both Ang-1 and VEGF concentrations have been reported to be significantly lower in patients with sepsis than in controls, but Ang-2 levels are higher and are associated with disease severity [18,19]. PDGF stimulation of vascular smooth muscle cells leads to a decrease in Ang-2 levels [20]. Elevated Ang-2 levels have been reported in severe sepsis and septic shock and may contribute to sepsis-related capillary leak [19,21-23].

Clinical data from adult studies [24-28] support the association of elevated plasma growth factor concentrations with sepsis. Studies in children have demonstrated increased plasma VEGF concentrations in meningococcal sepsis [29] and community-acquired pneumonia [30], and increased plasma PDGF and VEGF in respiratory syncytial virus infection [31], but these three growth factors together with Ang-1 and Ang-2 have never previously been explored in a large study in children. Given that the angiogenic factors have been identified as predictors of disease severity in sepsis, we aimed to determine whether the five angiogenic factors (PDGF, VEGF, FGF, and Ang-1 and Ang-2) may be mortality indicators in a population with a high burden of parasitic and HIV infection. We also aimed to investigate whether evidence exists of a relation between intracerebral production of angiogenic factors and mortality in bacterial meningitis. We selected three growth factors and two angiopoietins in an attempt to understand whether they may play a role in the mobilization of endothelial progenitor cells in severe bacterial infection.

## Materials and methods

### Ethics statement

Ethical approval for this study was granted from The College of Medicine Research Committee (COMREC), Malawi, and The Liverpool School of Tropical Medicine Local Research Ethics Committee. Parents or guardians gave written informed consent for children to enter the study.

### Study population

The study was part of a larger prospective observational study investigating the genetic susceptibility to invasive pneumococcal disease in Malawian children [32]. This study was conducted at Queen Elizabeth Central Hospital (QECH) in Blantyre, Malawi, between April 2004 and October 2006. We recruited children aged between 2 months and 16 years with a suspected diagnosis of bacterial meningitis or pneumonia. Details on enrolment criteria, laboratory methods, and management protocols were described elsewhere [33]. We also collected data on the duration of symptoms and on previous antibiotic administration. As our previous data indicated that these factors did not influence outcome in multivariate analysis, we did not include them in the analysis reported here [33]. We recorded the Blantyre Coma Score (BCS) on admission [34]; this has a scale from 0 to 5, with a score of ≤2 defining coma. We assessed each child's nutritional status by using weight-for-height Z scores and height-for-age Z scores. In total, we recruited 377 children to the parent study, but angiogenic factor determination was performed on only the first 293 cases, who constituted the study population of the present investigation. Pneumococcal bacterial loads were determined as previously described [33].

We used the following definitions:

*Cases *(*n* = 293): Children first seen with signs and symptoms of bacterial meningitis or pneumonia in whom growth factors were determined. 

*Healthy controls *(*n* = 15): Healthy afebrile children from the same villages as the cases, who had no malarial parasites on blood film. Controls were selected by parents or guardians in the neighborhood of the index case as part of a larger study investigating genetic susceptibility in IPD [32]. In a small number of children, parental consent also was given to take venous samples for cytokine and angiogenic factor determination. 

*Invasive pneumococcal disease (IPD) *(*n* = 180): *S. pneumoniae *was identified (by culture, microscopy, and Gram stain, antigen testing, or PCR) from one or more of the following normally sterile body sites: blood, cerebrospinal fluid, lung aspirate.

*Serious bacterial infection (SBI) *(*n* = 216): Children with bacterial meningitis or pneumonia, and in whom a bacterial pathogen was identified by culture, polysaccharide antigen test, or PCR in blood, cerebrospinal fluid or lung aspirate fluid (*Streptococcus pneumoniae, Neisseria meningitidis, and Haemophilus influenzae b*).

*No detectable bacterial infection (NBI) *(*n* = 77): Children with bacterial meningitis or pneumonia, but who were negative for any bacteria on culture, polysaccharide antigen test, or PCR (*S. pneumoniae, N. meningitidis, and H. influenzae b*). 

*Pneumonia *(*n* = 82): Confirmed by radiology and positive blood or lung aspirate by culture or PCR. 

*Bacterial meningitis *(*n* = 211): Confirmed by CSF cell count (>10 per microliter) and one of the following tests: CSF culture, Gram stain, polysaccharide antigen, or PCR positive.

### Growth factor, Ang-1, and Ang-2 determination

Growth-factor determination was performed in plasma and CSF samples by using Luminex 100 technology in the Bio-plex Protein Array System (Bio-Rad Laboratories. Inc., Santa Clara, California, USA) by using a 27-plex Bioplex Human Cytokine kit, which includes IL-1β, IL-1ra, IL-2, IL-4, IL-5, IL-6, IL-7, IL-8, IL-9, IL-10, IL-12 (p70), IL-13, IL-15, IL-17, eotaxin, basic FGF, G-CSF, GM-CSF, IFN-γ, IP-10, MCP-1 (MCAF), MIP-1α, MIP-1β, PDGF-BB, RANTES, TNF-α, and VEGF (Bio-Rad Laboratories), according to the manufacturer's instructions. In 50 children with bacterial meningitis, in whom sufficient CSF existed for analysis, CSF growth factors were determined on admission. Plasma Ang-1 and Ang-2 were determined by using a commercial ELISA assay (R&D Systems Europe, Ltd., Abingdon, UK). We have previously reported the analysis of chemokines and pro-and antiinflammatory cytokines in this cohort [33,35].

### HIV determination

HIV status was assessed in children 18 months or older by using at least two of the following tests; Unigold and Serocard (Trinity Biotech, Wicklow, Ireland), or Determine-HIV (Abbott Laboratories, Springfield, IL, USA). At least two tests were required to be positive for a subject to be classified as HIV infected. In children younger than 18 months, and in those with discordant antibody tests, HIV status was determined by using Amplicor HIV-1 DNA Test version 1.5 (Roche Diagnostics, South San Francisco, CA, USA).

### Statistical analysis

The growth factors and angiopoietins determined were summarized by using geometric means and interquartile ranges (IQRs). Two-sample *t *tests were used to compare growth-factor concentrations between groups, by using log-transformed data. Multiway analyses of variance were used to obtain adjusted comparisons for each factor of interest (main effects: SBI/NBI, pneumonia/meningitis, HIV status, survivor/nonsurvivor, and gram positive/negative infection). Correlations between growth factors and other variables were estimated by using Spearman's rho correlation coefficient. Fisher's Exact test was used to compare proportions. Univariable and multivariable logistic regression analyses were performed to develop a prognostic model of the influence of confounding factors (HIV status, age, sex, diagnosis, and previous antibiotics) on the primary outcome measure, inpatient mortality. CSF and plasma growth factors in children with bacterial meningitis were analyzed by using Wilcoxon's Signed Ranks test. Adjusted odds ratios (ORs) were obtained by using logistic regression. All tests were two-tailed, and a *P *value of < 0.05 was considered significant.

## Results

### Patient characteristics

We studied 293 children (57% boys), of whom 64 (22%) died. The median age was 2.4 years, and the IQR, 0.7 to 6.0 years. The 211 (72%) children were first seen with bacterial meningitis, and 82 (28%), with pneumonia; 154 (53%) children were HIV infected (50% of those with meningitis, and 60% of those with pneumonia). Baseline characteristics of study patients are shown in Table 1. In total, 216 (74%) children had a serious bacterial infection (SBI), and 77 had no organism identified (NBI). Of the 216 children with SBI, 182 (62%) had a gram-positive organism, 33 (11%) had a gram-negative organism, and one child had both gram-positive and -negative infections. The etiologies of both pneumonia and meningitis are shown in Table 2.

**Table 1:** Demographic, clinical, and laboratory characteristics of study patients by disease presentation

	Meningitis	Pneumonia	*P *value
No. of patients	211	82	

Age in years (median, IQR)	2.3 (0.6-6.0)	2.7 (0.9-5.6)	NS

Gender (male) (%)	116 (55%)	52 (63%)	NS

SBI (%)	176 (83%)	40 (49%)	0.0005

Gram-positive infection (%)^a^	146 (69%)	36 (44%)	NS

Gram-negative infection (%)^a^	29 (14%)	4 (5%)	NS

Blantyre Coma Score ≤2 (%)	88 (42%)	1 (1%)	0.0005

HIV infected (%)	105 (50%)	49 (60%)	NS

Duration of symptoms in days (median, IQR)	3 (2-4)	3 (3-6)	0.001

Inpatient mortality (%)	58 (28%)	6 (7%)	0.0005

Wasting (weight-for-height Z score ≤3 SD)	33/172 (19%)	7/69 (10%)	NS

Stunting (height-for-age Z score ≤3 SD)	31/206 (15%)	16/79 (20%)	NS

White cell count (×10^9^/L) (median, IQR)	11.8 (7.3-19.2)	15.7 (9.9-25.3)	0.001

C-reactive protein (mg/L) (median, IQR)	258 (162-323)	275 (56-345)	NS

Glucose (mmol/L) (median, IQR)	6.1 (4.8-7.6)	5.2 (4.4-6.0)	0.0005

Lactate (mmol/L) (median, IQR)	3.9 (2.4-6.3)	2.6 (1.8-5.2)	0.006

Systolic BP(mm Hg) (median, IQR)	103 (93-115)	100 (89-110)	NS

Diastolic BP(mm Hg) (median, IQR)	66 (59-80)	66 (59-76)	NS

^a^One child had mixed salmonella and pneumococcal infection (that is, mixed gram-positive and gram-negative infections). NS, not significant; *P *> 0.05.

**Table 2:** Etiology of pneumonia and meningitis

Organism	Meningitis	Pneumonia
*Streptococcus pneumoniae*	144	36

*Neisseria meningitidis*	10	0

*Salmonella enterica *serovar *Typhimurium*	5	1

*Salmonella enterica *serovar *Enteritidis*	2	0

*Haemophilus influenzae b*	7	3

*Haemophilus influenzae*	3	0

*Mixed S. enterica/S. pneumoniae*	1	0

Other (*E. coli, K. pneumoniae, S. pyogenes, S. aureus*)	4	0

Negative	35	42

Total	211	82

### Plasma VEGF, PDGF, and FGF in children with severe bacterial infection

Plasma VEGF, PDGF, and FGF on admission were significantly elevated in children with severe bacterial infection compared with healthy controls (Table 3). No significant difference in plasma growth factors was found between children with bacterial meningitis and those with pneumonia or between HIV-infected and HIV-uninfected children. The mean plasma VEGF concentrations were significantly higher in children with SBI compared with those with NBI, and plasma concentrations of all three growth factors were significantly higher in patients with gram-positive than in those with gram-negative infections (Table 3). Mean plasma PDGF concentrations were significantly higher in survivors compared with nonsurvivors. VEGF, PDGF, and FGF concentrations were significantly higher in children with invasive pneumococcal disease compared with children with SBI caused by pathogens other than *S. pneumoniae *(Table 3). PDGF concentrations were lower in children who had received antibiotics before hospital admission (*P *= 0.02). No significant differences were noted in mean VEGF, PDGF, and FGF concentrations in children with wasting or stunting and those without, and no correlation occurred with duration of symptoms (data not shown).

**Table 3:** Summary of growth factors in Malawian children with sepsis

Geometric mean (25%-75% centile) *P *values: univariable (multivariable^a^)	VEGF pg/ml	PDGF pg/ml	FGF pg/ml	Ang1 1,000 pg/ml	Ang2 1,000 pg/ml
Cases (*n* = 293)	90 (53-166)	956 (548-1884)	204 (119-376)	5.54 (2.6-9.7)	8.5 (5.2-13.6)
Controls (*n* = 15)	11 (4,15) *P *< 0.001	402 (195-721) *P *= 0.04	34 (20-48) *P *< 0.001	6.84 (2.2-20.1) *P *= 0.58	2.4 (1.6-4.6) *P *< 0.001

NBI (*n* = 77)	77 (49-133)	1,069.4 (702-2,309)	210 (141-342)	8.4 (3.9-16.8)	5.3 (3.1-7.6)
SBI (*n* = 216)	96 (56-171) *P *= 0.06 (0.02)	918 (521-1,779) *P *= 0.27 (0.71)	202 (107-89) *P *= 0.75 (0.74)	4.7 (2.4-8.7) *P *< 0.001 (0.01)	10.0 (5.8-16.1) *P *< 0.001 (0.002)

Gram-positive infection (*n* = 182)	102 (59-181)	978 (574-1,817)	215 (118-404)	4.8 (2.5-8.9)	10.4 (6.1-16.8)
Gram-negative infection (*n* = 33)	63 (40-96) *P *= 0.004 (0.01)	643 (286-1,616) *P *= 0.03 (0.03)	134 (84-278) *P *= 0.007 (0.004)	4.2 (1.8-8.6) *P *= 0.41 (0.54)	9.1 (5.7-14.2) *P *= 0.23 (0.61)

Pneumonia (*n* = 211)	88 (54-155)	900 (528-1,756)	193 (116-356)	5.1 (2.5-9.6)	9.0 (5.3-15.8)
Meningitis (*n* = 82)	97 (51-200) *P *= 0.38 (0.19)	1114 (676-2,378) *P *= 0.11 (0.49)	239 (131-404) *P *= 0.06 (0.15)	6.9 (3.7-14.6) *P *= 0.10 (0.63)	7.0 (5.2-8.7) *P *= 0.01 (0.52)

HIV negative (*n* = 138)	84 (53-145)	937 (547-1,720)	201 (117-377)	5.4 (2.7-9.7)	6.4 (3.9-9.2)
HIV positive (*n* = 154)	96 (53-177) *P *= 0.17 (0.45)	967 (569-2,035) *P *= 0.79 (0.95)	208 (123-375) *P *= 0.71 (0.70)	5.7 (2.5-10.0) *P *= 0.91 (0.72)	10.9 (6.2-16.8) *P *< 0.001 (<0.001)

Survivors (*n* = 229)	93 (42-142)	1,051 (361-1,261)	214 (115-314)	6.0 (2.8-10.2)	7.7 (5.0-12.6)
Nonsurvivors (*n* = 64)	81 (54-176) *P *= 0.27(0.19)	682 (612-2,035) *P *= 0.003 (0.007)	171 (119-385) *P *= 0.07 (0.10)	3.9 (2.3-7.4) *P *= 0.03 (0.03)	11.9(6.7-21.7) *P *= 0.001 (0.02)

Invasive pneumococcal disease (IPD) (*n* = 180)	101 (58-181)	978 (593-1,818)	215 (119-397)	4.8 (2.5-8.7)	10.4 (6.1-16.8)
SBI, other than IPD (*n* = 35)	68 (41-115) *P *= 0.01	671 (292-1,611) *P *= 0.04	146 (86-308) *P *= 0.02	4.6 (2.0-9.1) *P *= 0.32	8.3 (5.6-13.0) *P *= 0.16

^a^Multivariable analyses included NBI/SBI, diagnosis(pneumonia/meningitis), HIV status, survival status and gram-positive/-negative type. (IPD/SBI, other than IPD was not included in the model because of strong association with gram-positive/negative status. All except two of gram-positive infections were IPD).

### CSF VEGF, PDGF, and FGF in children with bacterial meningitis

**Figure 1:**
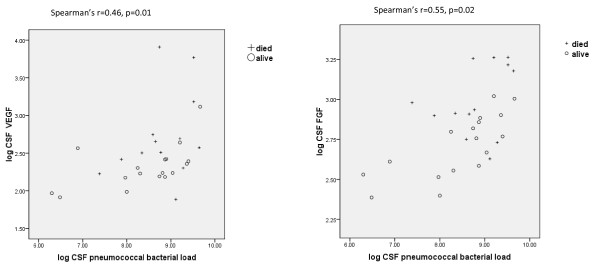
**Scatterplot showing CSF VEGF and FGF against CSF pneumococcal bacterial load in children with pneumococcal meningitis**.

In 50 children with bacterial meningitis, CSF VEGF, PDGF, and FGF were measured. CSF concentrations of VEGF, PDGF, and FGF were significantly higher than paired plasma concentrations (*P *= 0.001; *P *< 0.005; and *P *< 0.0005, respectively, Wilcoxon signed rank test). No significant correlations appeared between the CSF concentrations of VEGF, PDGF, or FGF and the CSF white cell count, CSF absolute neutrophil count, or Blantyre coma score. In children with pneumococcal meningitis (*n* = 30), significant correlations were noted between CSF pneumococcal bacterial load and the concentration of VEGF and FGF in the CSF (Figure 1), and the CSF concentrations of both of these growth factors were higher in patients who died than in those who survived.

No significant differences were found in CSF VEGF, PDGF, and FGF levels between children with coma (BCS ≤2) and those without. In contrast to plasma concentrations, mean CSF, VEGF, and FGF concentrations were higher in nonsurvivors than in survivors (1,178 versus 216 pg/ml; *P *= 0.02; and 939 versus 501 pg/ml; *P *= 0.001, respectively).

### Plasma Ang-1 and Ang-2 in children with severe bacterial sepsis

Plasma Ang-2 on admission was significantly increased in children with severe bacterial infection compared with healthy controls, but Ang-1 was not significantly different (Table 3). No significant differences in Ang-1 and Ang-2 concentrations were noted between children with meningitis and those with pneumonia, but Ang-2 was significantly elevated in HIV-infected children. The mean plasma Ang-1 concentrations were significantly lower in children with SBI compared with those with NBI, but Ang-2 was significantly higher after adjustment for confounding variables. Ang-1 and Ang-2 plasma concentrations were not significantly different between gram-positive and gram-negative infections (Table 3). Mean plasma Ang-1 concentrations were significantly higher, and Ang-2, significantly lower in survivors compared with nonsurvivors (Table 3). The ratio of lnAng-2 (natural log Ang-2) to lnAng-1 was higher in nonsurvivors compared with survivors (*P *= 0.03). Plasma Ang-1 concentrations were not significantly different in children with invasive pneumococcal disease compared with children with SBI caused by pathogens other than *S. pneumoniae *(Table 3). Plasma Ang-2 correlated positively with the pro- and antiinflammatory cytokines, IL-1Ra, IL-6, IL-8, and IL-10 (Table 4).

**Table 4:** Correlation between plasma angiogenic factors and pro- and antiinflammatory cytokines

	Plasma IL-1Ra (pg/ml)	Plasma IL-6 (pg/ml)	Plasma IL-8 (pg/ml)	Plasma IL-10 (pg/ml)
Plasma VEGF (pg/ml)	NS	NS	0.22 *P *< 0.0005	0.37 *P *< 0.0005

Plasma PDGF (pg/ml)	-0.16 *P *= 0.06	NS	NS	NS

Plasma FGF (pg/ml)	NS	NS	0.13 *P *= 0.03	0.38 *P *< 0.0005

Plasma Ang-1 (pg/ml)	-0.37 *P *< 0.0005	-0.26 *P *< 0.0005	NS	NS

Plasma Ang-2 (pg/ml)	0.53 *P *< 0.0005	0.44 *P *< 0.0005	0.50 *P *< 0.0005	0.34 *P *< 0.0005

NS, not significant.

### Logistic regression models for predicting mortality and SBI

The plasma values of VEGF, PDGF, FGF, Ang-1, and Ang-2 were log transformed and included in a multivariate stepwise logistic regression model, including HIV status, sex, diagnosis (pneumonia or meningitis), and admission lactate, as variables in the equation. Female sex (OR, 3.95; 95% CI, 1.33 to 11.76), and Ang-1 (OR, 0.23; 95% CI, 0.08 to 0.69) were significantly associated with mortality. By using a similar model, meningitis (OR, 5.91; 95% CI, 1.47 to 23.77), admission lactate (OR, 3.20; 95% CI, 1.20 to 8.57), VEGF (OR, 5.63; 95% CI, 1.32 to 24.11), Ang-1 (OR, 0.19; 95% CI, 0.06 to 0.62), and Ang-2 (OR, 5.40; 95% CI, 1.79 to 16.30) were significantly associated with SBI (Table 5).

**Table 5:** Multivariate logistic regression model to predict mortality and SBI

Predictor	Adjusted OR for death (95% CI)	*P*	Adjusted OR for SBI (95% CI)	*P*
Female sex	3.95 (1.33-11.76)	0.01	1.59 (0.47-5.42)	0.46

Meningitis	9.9 × 10^12^	1.0	5.91 (1.47-23.77)	0.01

HIV uninfected	0.60 (0.18-1.97)	0.4	1.18 (0.34-4.13)	0.8

Admission lactate	1.33 (0.52-3.37)	0.6	3.20 (1.20-8.57)	0.02

Plasma FGF (pg/ml)	1.35 (0.52-3.54)	0.5	0.94 (0.31-2.91)	0.92

Plasma VEGF (pg/ml)	1.21 (0.43-3.42)	0.7	5.63 (1.32-24.11)	0.02

Plasma PDGF (pg/ml)	3.17 (0.90-11.12)	0.07	1.07 (0.35-3.28)	0.91

Plasma Ang-1 (pg/ml)	0.23 (0.08-0.69)	0.009	0.19 (0.58-0.62)	0.006

Plasma Ang-2 (pg/ml)	0.90 (0.45-1.83)	0.6	5.40 (1.79-16.29)	0.003

## Discussion

Our study examined both growth factors and angiogenic factors in 293 children and demonstrates that among Malawian children with severe bacterial infection, high plasma VEGF, PDGF, FGF, and Ang-1 concentrations are associated with a favorable outcome. In contrast, high Ang-2 concentrations are associated with an unfavorable outcome. In children with bacterial meningitis, our data suggest intracerebral production of angiogenic factors, and an association between high intrathecal concentrations and mortality. Inpatient mortality is high in children admitted with pneumonia and bacterial meningitis in Malawi; therefore, it is important to determine the utility of these angiogenic factors as biomarkers for the identification of patients at risk of a poor outcome.

Our data are in keeping with current evidence that suggests that the growth factors together with Ang-1 may be involved in limiting the deleterious effects of sepsis-induced endothelial dysfunction. Consistent with previous work, growth-factor concentrations were significantly higher in cases compared with controls. In contrast to previous studies, which demonstrated highest levels of growth factors in patients with septic shock [24,25,27,29], we showed that levels were lower in those with the most severe disease, defined as having a fatal outcome. Very few of our patients demonstrated septic shock or required aggressive fluid resuscitation. Our data are consistent with those of Brueckmann *et al. *[26], who demonstrated that adults with PDGF levels <200 pg/ml were 7 times more likely to die than were those with higher levels.

Karlsson *et al. *[28] demonstrated that VEGF concentrations in adult patients with sepsis were lower in nonsurvivors than in survivors, but did not adequately predict mortality. The differences in growth factors between gram-positive and gram-negative infections are difficult to explain. Our study was not designed to explain this differential response. We speculate that differences in the way bacterial cell components stimulate the inflammatory cascade might be responsible. We identified high plasma Ang-1 concentrations and male gender as being independently associated with survival. Our study also supports the concept of intracerebral production of growth factors in bacterial meningitis.

The major limitation of our study was that we studied growth-factor and angiopoietin concentrations only at admission and did not follow their course over time. Admission values are potentially more useful as prognostic markers, if they can be made available to the clinician at the time the patient is first seen, as they could help to identify a group of patients requiring aggressive treatment or characterize those eligible for entry to a randomized clinical trial of adjunctive therapies.

Interventions that target the inhibition of inflammatory mediators and coagulation pathways have been unsuccessful. Recently, microcirculatory dysfunction has been shown to be a critical element of the pathogenesis of severe sepsis [36]. The investigation of host mediators that directly influence endothelial function might therefore be a valuable approach to improve our understanding of the pathophysiology of sepsis.

A recent study demonstrated that activated protein C (APC) uses the angiopoietin/Tie-2 axis to promote endothelial barrier function [37]. Large clinical trials with APC showed a beneficial effect in adult patients with severe sepsis [38], but in children, this effect was not seen [39]. Assessment of the angiopoietin/Tie-2 system might help to identify those children who might benefit from APC therapy or other new adjunctive therapies.

Our study contributes to the understanding of factors controlling endothelial integrity, and our results are consistent with those of previous studies [19,22,26,28]. Although the number of controls in our study was small, the inclusion of a comparator group allows the assessment of possible effects of other asymptomatic coinfections, such as helminths and malaria parasitemia. Three studies in children have reported increased Ang-2 concentrations in severe malaria [40,41] and cerebral malaria [41,42]. A recent study from Thailand [41] reported that Ang-1 and Ang-2 discriminated severe from uncomplicated malaria, and Ang-1 distinguished children with severe malaria from those with cerebral malaria. The authors propose that Ang-1 and Ang-2 are attractive candidates for a point-of-care test to identify individuals with a risk of progression to severe disease, as they can be incorporated into rapid lateral-flow immunochromatographic tests such as those used in malaria diagnosis.

As our patient population differs significantly from those of most of the readers of this journal, inferences regarding other study populations may be difficult to make. Nonetheless, we believe that the high mortality in our patients represents the most severe end of the spectrum (that is, MODS without intensive care support), which ultimately results from severe endothelial dysfunction. Although our study does not provide any description of multiorgan dysfunction, data from studies in similar settings suggest that in severely ill children without malaria, Blantyre Coma Score [43,44] and lactate [44] accurately predict mortality.

The mobilization of endothelial progenitor cells (EPCs) from bone marrow to sites of endothelial injury is induced by angiogenesis. A recent study demonstrated that the number and function of EPCs decreased in the progression of sepsis and may be one of the main pathogenic factors in multiple organ dysfunction syndromes [5]. EPCs are increased in the blood of patients with sepsis, in parallel with VEGF levels [45]. Our data support the concept that the angiogenic factors reported here are important in the pathophysiology of severe bacterial infection.

Studies investigating host responses to infection have shown that most mediators are increased and are positively associated with disease severity. We previously showed that the concentration of the chemokine, Regulated on Activation Normal T Cells Expressed and Secreted (RANTES) is inversely associated with disease severity in children, both in meningococcal disease [46] and in pneumococcal disease [35]. Others confirmed this finding in meningococcal disease [47]. This study now adds another group of cytokines and angiopoietins that are inversely associated with disease severity.

VEGF has been shown to be elevated in the CSF of children and adults with bacterial meningitis [48] and in adults with cryptococcal meningitis [49]. Both studies suggest that the VEGF is produced intrathecally and may contribute to the blood-brain barrier disruption. Our data would also be consistent with intrathecal production of growth factors. We demonstrated higher CSF than plasma concentrations in paired samples, and higher concentrations of VEGF and FGF in the CSF of children who died. We previously reported data that suggest a compartmentalized host response in pneumococcal meningitis [33]. In contrast to the study by van der Flier [48], we found no association between the CSF growth factors and the CSF white cell count.

Neutrophils have been shown to secrete VEGF in response to pneumococcal stimulation, and we suggest that VEGF may play a role as a mediator of vascular permeability [50]. VEGF and PDGF are working in a complex relation with Ang-1 and Ang-2 to promote endothelial cell survival and to prevent apoptosis [17]. Our data showing a favorable outcome in children with higher plasma levels of FGF, VEGF, PDGF, and Ang-1 would be consistent with this theory. Ang-2 appears to be acting as an antagonist to the other angiogenic factors and correlates positively with disease severity. The dysregulation of Ang-1 and Ang-2 in severe sepsis may contribute to the endothelial dysfunction and increased vascular permeability that lead to multiorgan failure and mortality.

## Conclusions

We have shown that low plasma VEGF, PDGF, FGF, and Ang-1 concentrations are associated with an unfavorable outcome in children with severe bacterial infection, the association being independent of confounding factors in the case of Ang-1. High Ang-2 concentrations are associated with mortality. In bacterial meningitis, our data support the concept of intracerebral production of growth factors, with increased CSF concentrations in nonsurvivors. VEGF, PDGF, FGF, Ang-1, and Ang-2 may be key players in the endothelial dysregulation seen in severe bacterial infection, or they may simply reflect an attempt by the host to repair endothelial damage. The measurement of these five factors might be useful (a) as prognostic markers of outcome, and (b) in identifying children who might benefit from adjunctive new therapies. Further studies are needed to identify the exact mechanism by which the angiopoietins might affect endothelial function in severe bacterial infection.

## Key messages

• Mean VEGF, PDGF, and FGF concentrations are higher in survivors than in nonsurvivors.

• Mean Ang-1 is significantly increased, and Ang-2 significantly decreased, in survivors compared with nonsurvivors.

• Low Ang-1 is independently associated with mortality.

• In bacterial meningitis, mean CSF VEGF, PDGF, and FGF concentrations were higher than paired plasma concentrations, and mean CSF VEGF and FGF concentrations were higher in nonsurvivors than in survivors.

• Ang-1 could be a useful prognostic marker.

## Abbreviations

Ang-1: angiopoietin-1; Ang-2: angiopoietin-2; FGF: fibroblast growth factor; IPD: invasive pneumococcal disease; NBI: no detectable bacterial infection; PDGF: platelet-derived growth factor; SBI: serious bacterial infection; VEGF: vascular endothelial growth factor.

## Competing interests

The authors declare that they have no competing interests.

## Authors' contributions

EDC designed the study, recruited patients, performed data analysis, and drafted the manuscript. CAH, MEM, and EMM were involved in study design and drafting the manuscript. LAM recruited patients and helped draft the manuscript. IPD Study Group recruited patients. DLB, GJ, PB, SN, and HP performed laboratory analysis and helped draft the manuscript. SW provided statistical advice and helped with data analysis. RSH helped draft the manuscript.
